# Frequency-Dependent Multi-Well Cardiotoxicity Screening Enabled by Optogenetic Stimulation

**DOI:** 10.3390/ijms18122634

**Published:** 2017-12-06

**Authors:** Susanne Rehnelt, Daniela Malan, Krisztina Juhasz, Benjamin Wolters, Leo Doerr, Matthias Beckler, Ralf Kettenhofen, Heribert Bohlen, Tobias Bruegmann, Philipp Sasse

**Affiliations:** 1 Institute of Physiology I, Medical Faculty, University of Bonn, 53127 Bonn, Germany; s4surehn@uni-bonn.de (S.R.); dmalan@uni-bonn.de (D.M.); 2 Nanion Technologies GmbH, 80636 Munich, Germany; krisztina.juhasz@nanion.de (K.J.); leo.doerr@nanion.de (L.D.); matthias.beckler@nanion.de (M.B.); 3 Present address: Institute for Nanoelectronics, Department of Electrical Engineering and Information Technology, Technische Universität München, 80339 Munich, Germany; 4 Part of the Ncardia Group, Axiogenesis AG, 50829 Cologne, Germany; benjamin.wolters@ncardia.com (B.W.); ralf.kettenhofen@ncardia.com (R.K.); heribert.bohlen@ncardia.com (H.B.); 5 Research Training Group 1873, University of Bonn, 53127 Bonn, Germany

**Keywords:** optogenetics, field potential, long QT syndrome, cardiotoxicity screening, heart rate, cardiomyocytes, induced pluripotent stem cells

## Abstract

Side effects on cardiac ion channels causing lethal arrhythmias are one major reason for drug withdrawals from the market. Field potential (FP) recording from cardiomyocytes, is a well-suited tool to assess such cardiotoxic effects of drug candidates in preclinical drug development, but it is currently limited to the spontaneous beating of the cardiomyocytes and manual analysis. Herein, we present a novel optogenetic cardiotoxicity screening system suited for the parallel automated frequency-dependent analysis of drug effects on FP recorded from human-induced pluripotent stem cell-derived cardiomyocytes. For the expression of the light-sensitive cation channel Channelrhodopsin-2, we optimised protocols using virus transduction or transient mRNA transfection. Optical stimulation was performed with a new light-emitting diode lid for a 96-well FP recording system. This enabled reliable pacing at physiologically relevant heart rates and robust recording of FP. Thereby we detected rate-dependent effects of drugs on Na^+^, Ca^2+^ and K^+^ channel function indicated by FP prolongation, FP shortening and the slowing of the FP downstroke component, as well as generation of afterdepolarisations. Taken together, we present a scalable approach for preclinical frequency-dependent screening of drug effects on cardiac electrophysiology. Importantly, we show that the recording and analysis can be fully automated and the technology is readily available using commercial products.

## 1. Introduction

Since 1953, over 60 drugs have had to be withdrawn from the market because of the risk of cardiac arrhythmia [1] leading to sudden cardiac death [2]. Therefore, better methods are required to enhance the quality and power of preclinical safety screenings [3] to ensure patients’ safety and to save billions of dollars because of late-stage drug withdrawals [4,5,6]. One of the most common cardiotoxic effects is delayed cardiac repolarisation, which clinically manifests in the electrocardiogram (ECG) as a prolonged QT interval and which is strongly associated with lethal ventricular tachyarrhythmias of the Torsades de Pointes type [2]. Therefore, the International Conference on Harmonization’s guideline S7B established rules for assessing the cardiac safety of newly developed drugs before entering into clinical trials [7]. This guideline focuses on in vitro experiments measuring the rapid potassium current (I_Kr_) through the *human Ether-a-go*-*go Related Gene* (hERG) channel expressed in heterologous cell lines, which was considered to be the best surrogate measurements for delayed repolarisation [7], because the inhibition of hERG channels is responsible for most instances of drug-induced Long QT syndrome [5,8,9]. However, screening systems relying on measuring one type of ion channels do not take into account side effects on other cardiac ion channels, which can also be highly proarrhythmic. For instance, inhibiting Ca^2+^ channels lead to faster repolarisation and short QT syndromes, and blocking Na^+^ channels results in slowed and dispersed electrical conduction and Brugada syndromes, which can both be highly proarrhythmic [2,5,8,10]. Furthermore, it is possible that drugs affecting multiple ion channels cause several effects which neutralise each other and such drugs would be unnecessarily rejected when relying on a single channel assay [11]. Thus, in contrast to heterologous expression systems, intact human cardiomyocytes with all channels present in their native environment would be the best suited screening system for analysing cardiotoxic side effect of drugs. To take these considerations into account, the “comprehensive in vitro proarrhythmia assay” (CiPA) initiative was launched in 2013 with the aim to validate novel cardiotoxicity screening systems towards a multimodal assessment of various cardiac ion channels. One of the CiPA demands was to analyse drug effects on human cardiomyocytes [9]. For this purpose, human-induced pluripotent stem cell (hiPSC)-derived cardiomyocytes provide a cost-efficient and reproducible source with high similarity in ion channel composition to adult human cardiomyocytes [12,13].

The manual patch clamp is the gold standard technique to investigate the electrophysiology of cardiomyocytes, but is also very time consuming and the duration of experiments is limited due to rundown of cytosolic stability [14]. Analysis of the electrical activity by recording extracellular field potentials (FP) with microelectrode arrays (MEA) is a non-invasive and well-suited alternative enabling high throughput assays [15]. FP signals reflect the action potential (AP) shape, making it possible to extract relevant AP parameters: The initial rapid component of the FP correlates to the AP upstroke velocity and quantification by measuring the maximum negative downstroke velocity (max.DV) is a well-suited surrogate parameter for Na^+^ channel activity [16,17]. In addition, the FP consists of a deflection at the end similar to the T-wave in the human ECG. This allows the straightforward determination of field potential duration (FPD), which is highly correlated to the AP duration (APD) [16]. Thus, parameters analysed from FP recordings make it possible to assess the cardiac safety of drugs. Therefore, recording FP with MEA has gained more and more importance in the last decade [5,6,16,18,19,20,21,22,23,24,25,26]. This is also due to its easy-to-use commercially available instrumentation, as well as the very high temporal resolution, which is in contrast to voltage imaging technologies that require dye-loading, high-intensity illumination and custom-built recording devices [27,28,29]. However, the FP recording technology still faces limitations towards industrial cardiotoxicity screening in pharmaceutical companies. Automated analysis is often not possible because FP recorded from small individual electrodes show severe variations in FP shape, requiring manual selection and biased analysis by investigators [6,15]. Using larger electrodes (>500 μm) that record one FP from thousands of cardiomyocytes simultaneously leads to robust FP signals with lower variation between electrodes. However, the non-synchronous electrical activity due to the conduction through the electrical syncytium on a large electrode results in fragmented signals during the initial rapid fast component of the FP, which prevents proper analysis of the Na^+^ channel availability [30]. The second important limitation is that FP recordings are restricted to the spontaneous activity of cardiomyocytes because electrical stimulation is technically very challenging and induces large stimulation artefacts [31,32,33]. However, the effects of drugs on cardiac ion channels can be strongly dependent on the beating rate due to intrinsic biophysical properties of the proteins and the use-dependency of drugs [34,35]. Importantly, better differentiation and selection protocols to obtain more mature hiPSC-derived cardiomyocytes are emerging [36,37,38], which will ultimately result in electrical silent ventricular-like cardiomyocytes with stable resting potential, which will make pacing indispensable.

To overcome these limitations, we recently combined optogenetic stimulation and FP recordings on MEA. We used brief blue light (470 nm) pulses to reliably pace Channelrhodopsin-2 (ChR2) expressing hiPSC-derived cardiomyocytes at physiological heart rates from 1 to 2.5 Hz (60 to 150 bpm). In addition, global illumination of cardiomyocytes on all 60 recording electrodes of one multi-electrode MEA induced simultaneous electrical activity without any detectable conduction delay. This allowed spatial averaging of FP from all electrodes resulting in a robust FP shape similar to using one large electrode [30], but without fragmentation of the initial fast rapid component. Using this technology, we were able to analyse FPD and max.DV and thereby detected frequency-dependent drug effects on Na^+^, Ca^2+^ and K^+^ channels [17]. However, these experiments were performed in a low throughput format using a single-well MEA system and a custom designed lens-based optogenetic stimulation that does not allow upscaling, as required for industrial drug screening.

Therefore, in this report, we combined optogenetic pacing of hiPSC-derived cardiomyocytes with a commercially available 96-well screening platform (CardioExcyte96, Nanion Technologies, Munich, Germany) equipped with one large FP recording electrode per well [30] to enable fully automated frequency-dependent cardiotoxicity screening. For this purpose, we optimised ChR2 mRNA- or virus-based gene transfer and developed light-emitting diode (LED) lids for exact optogenetic stimulation of hiPSC-derived cardiomyocytes at physiological heart rates from 1 to 3 Hz (60 to 180 bpm). Using specific ion channel blockers, we confirmed the cardiac ion channels involved in the different parts of the FP and applying known arrhythmogenic drugs, we proved the concept for cardiotoxicity screening. Importantly, we demonstrate that this technology can be performed with commercially available products and can be fully automated.

## 2. Results

### 2.1. Establishing LED-Based Optogenetic Pacing on 96-Wells Plates

**Figure 1:**
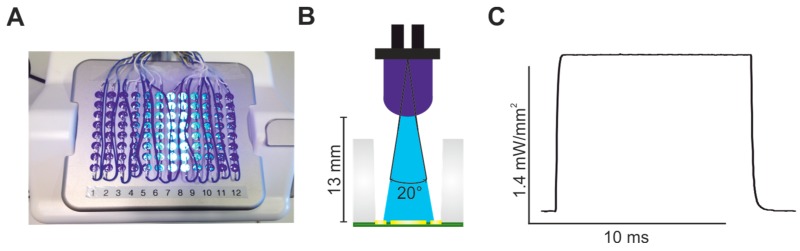
Custom-built prototype of a LED lid for the CardioExcyte96 system. (**A**) Picture of the lid containing 96 blue LEDs; (**B**) sketch of a single well with one LED placed 13 mm above the recording electrode (yellow) with an illumination angle of 20°; (**C**) light intensity of a 10 ms long light pulse at the level of the recording electrode.

The CardioExcyte96 system is an extracellular FP and impedance amplifier using a 96-well plate with one gold recording electrode of 2 mm diameter in the centre of each well and one reference electrode at the side [30]. To globally illuminate every single well of the 96-well CardioExcyte sensor plate, we custom built an LED lid containing 96 high-power 470 nm LEDs (NSPB500AS, Nichia, Tokushima, Japan) with a radiation angle of ~20° to ensure illumination of the whole recording electrode in each well (Figure 1A,B). LEDs were driven with short constant current pulses (100 mA), resulting in a light intensity of 1.4 mW/mm^2^ at the cell level. Maximum light intensity was reached in less than 40 μs (Figure 1C), allowing efficient stimulation with precise light pulses in the millisecond range.

### 2.2. Maturation and Optical Pacing of Cardiomyocytes

**Figure 2:**
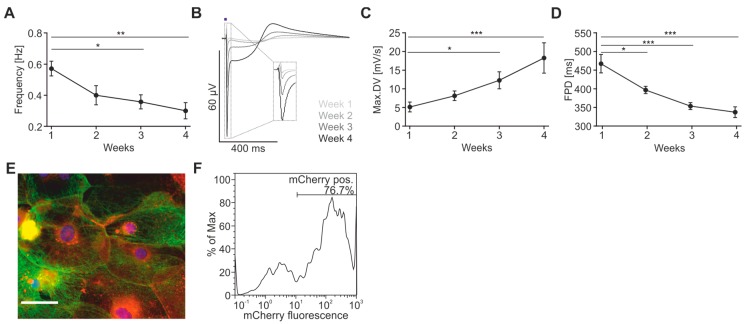
Analysis of field potential (FP) signals over four weeks after adeno-associated virus (AAV)-transduction and plating of human-induced pluripotent stem cell (hiPSC)-derived cardiomyocytes. (**A**) Spontaneous beat rate over time (from week 1 to week 4: −47 ± 17%; *p* = 0.0077; *n* = 7); (**B**) overlay of representative FP optically paced at 1 Hz (blue bar); (**C**) analysis of maximum negative downstroke velocity (max.DV) (from week 1 to week 4: +256 ± 22%; *p* = 0.0005; *n* = 7) and (**D**) field potential duration (FPD) over time (from week 1 to week 4: −28 ± 4%; *p* < 0.0001; *n* = 7); (**E**) microscope picture of Channelrhodopsin-2 (ChR2)-expressing Cor.4U^®^ cells taken at week 3 (blue = DAPI, green = Troponin T, red = mCherry from ChR2-mCherry fusion protein, scale bar: 50 μm); (**F**) representative (*n* = 3) fluorescence-activated cell sorting (FACS)-profile of ChR2-mCherry positive cells at week 3. * *p* < 0.05, ** *p* < 0.01, *** *p* < 0.001.

Commercially available hiPSC-derived cardiomyocytes (Cor.4U^®^, Ncardia, Cologne, Germany) were transduced with an adeno-associated virus (AAV) to express the light-gated non-selective cation channel ChR2 [39] in fusion to the red fluorescent protein mCherry. One day after infection, cells were plated on the recording electrodes of the CardioExcyte sensor plates where they formed a spontaneously beating confluent monolayer. The FP were recorded over four weeks and we observed electrophysiological “maturation” of the FP signals over time similar to a recent report using human cardiomyocytes differentiated from embryonic stem cells [40]. This became evident by a ~50% slowing of the spontaneous beating rate from week 1 to week 4 (Figure 2A). In addition, we characterised the FP shape using 1 Hz optical stimulation (stimulation parameters see below) and found that the max.DV increased by 256% over time (Figure 2B,C). This is in line with a recent report and indicates higher Na^+^ channel expression [40]. Furthermore, we found a shortening of the FPD over time from week 1 to week 4 by 28%, and a more pronounced repolarisation wave (Figure 2B,D) which could be explained by a higher level of repolarisation K^+^ currents or better electrical synchronisation of cardiomyocytes. 

Thus, in this study, we measured FP signals three to four weeks after *ChR2* gene transfer and cardiomyocyte plating on the CardioExcyte sensor plates. To prove that the single AAV-based gene transfer was still sufficient, we assessed ChR2 expression and function three weeks after infection. Cardiomyocytes showed membrane bound ChR2 expression (Figure 2E) and fluorescence-activated cell sorting (FACS)-analysis revealed that 70 ± 11% (*n* = 3) of cells were positive for ChR2 (Figure 2F). 

### 2.3. Frequency Dependence of FP Parameters

**Figure 3:**
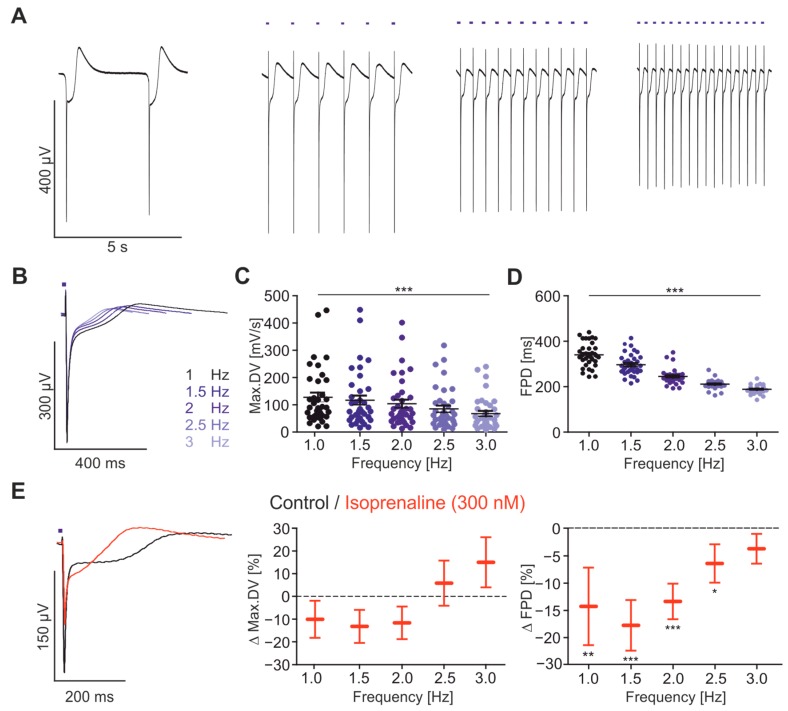
Optogenetically paced FP at various physiological stimulation rates. (**A**) Representative FP traces during (from left to right) spontaneous beating and optical pacing (blue bars) at 1 Hz, 2 Hz and 3 Hz; (**B**) overlay of representative FP paced at frequencies 1–3 Hz (from black to light blue); (**C**) statistical analysis of absolute values of max.DV (from 1 to 3 Hz: −47 ± 14%, *p* < 0.0001, *n* = 37) and (**D**) FPD (from 1 to 3 Hz: −43 ± 1%, *p* < 0.0001, *n* = 42); (**E**) Representative FP before and after application of 300 nM Isoprenaline and relative changes of max.DV (**middle**) and FPD (**right**) (*n* = 24). * *p* < 0.05, ** *p* < 0.01, *** *p* < 0.001.

We next tested the minimal requirement of light stimulation with the custom-built LED lid and found that light pulses (1.4 mW/mm^2^) as short as 2 ms were sufficient for reliable pacing at frequencies between 1 and 3 Hz (Figure 3A). In accordance to our previous study [17], we could detect frequency-dependent changes of the FP parameters. The max.DV decreased from 1 to 3 Hz by 47% (Figure 3B,C) and the FPD shortened from 1 to 3 Hz by 43% (Figure 3B,D). The latter finding is in full accordance with the physiological APD shortening measured in human cardiomyocytes [41] and QT shortening seen in the human ECG [42] at higher heart rates.

Adrenergic stimulation results in faster and stronger beating of the heart and shortening of AP through the G_s_-signalling pathway and phosphorylation of Ca^2+^ channels, ryanodine receptors and repolarising K^+^ channels. To detect this physiological effect with our approach, we applied the β-adrenergic agonist Isoprenaline (300 nM, Figure 3E, left). This increased the spontaneous beating rate from 0.25 ± 0.003 to 0.96 ± 0.11 Hz (*p* < 0.0001, *n* = 29) proving the positive chronotropic effect [5,43]. While Isoprenaline had no effect on the max.DV (Figure 3E, middle), it significantly reduced the FPD at all frequencies except at 3 Hz (Figure 3E, right), suggesting an augmentation of repolarising K^+^ currents.

### 2.4. Pharmacological Characterisation of FP Signals

**Figure 4:**
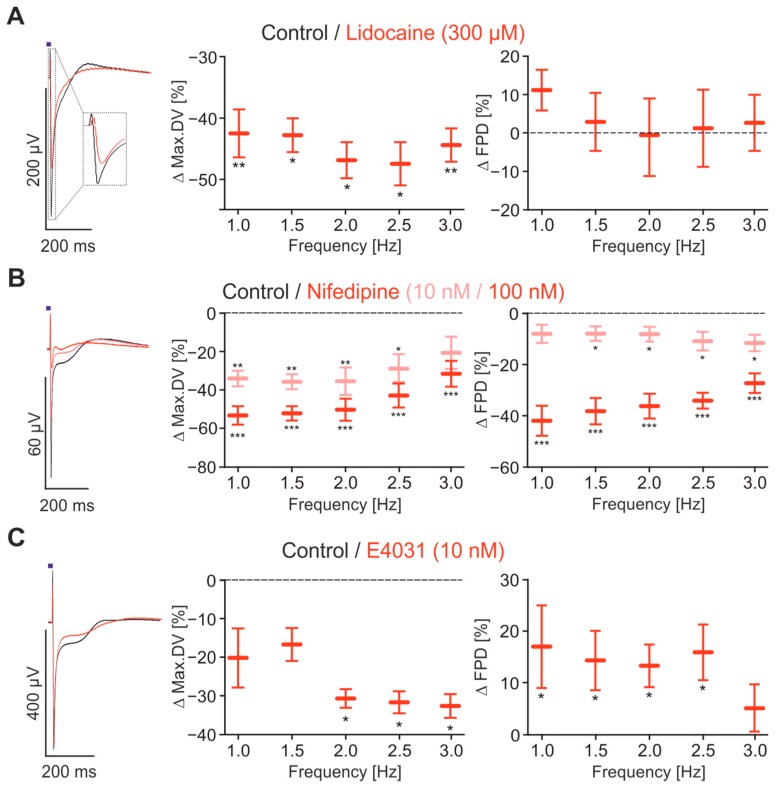
Pharmacological characterisation of FP parameters. (**A**–**C**) Overlay of representative FP optically paced at 1 Hz (**left**) before (black) and after drug application (red), and statistical analysis of the relative changes of max.DV (**middle**) and FPD (**right**) at the frequencies 1–3 Hz; (**A**) effects of 300 μM Lidocaine (*n* = 7–8); (**B**) effects of 10 nM (*n* = 7) and 100 nM (*n* = 11) Nifedipine; (**C**) effects of 10 nM E4031 (*n* = 12–17). * *p* < 0.05, ** *p* < 0.01, *** *p* < 0.001.

To prove that optogenetically paced FP signals allow the detection of specific effects on the three major cardiac ion channels, we tested Na^+^, Ca^2+^ and K^+^ current blockers at various physiological heart rates. Specifically, we applied the Na^+^ channel blocker Lidocaine (300 μM) and found a significant reduction of the max.DV parameter at all frequencies but, importantly, no unspecific side effects on the FPD (Figure 4A). Nifedipine, a highly specific Ca^2+^ channel blocker, reduced the max.DV at all pacing rates and additionally shortened the plateau phase of the FP and thereby reduced the FPD in a dose-dependent manner (Figure 4B). This data indicates that the max.DV parameter is not only influenced by Na^+^, but also by Ca^2+^ channel function. The latter can be explained by the rather immature status of the hiPSC-derived cardiomyocytes with elevated resting membrane potentials [44].

Interestingly, the effect of Nifedipine on FPD shortening was strongest at low heart rates and high concentrations (100 nM, Figure 4B right) nearly abolished the rate-dependency of FPD. This suggests that refractoriness of Ca^2+^ channels is one important mechanism for the shortening of FPD at higher heart rates in hiPSC-derived cardiomyocytes, which was described before [17,41]. To determine the effect of repolarising K^+^ currents, we applied E4031, which is a specific blocker for hERG K^+^ channels at low concentrations. While already using a very low dose of 10 nM E4031, we detected a delayed repolarisation phase with prolonged FPD, which is in line with recent reports [22,24,25,45]. However this effect was most prominent at slow beating rates and not present at 3 Hz pacing (Figure 4C). Furthermore, E4031 showed an unexpected side effect of reduced max.DV at high beating rates (Figure 4C), which could be explained by elevation of resting membrane potentials and subsequent inactivating of depolarising Na^+^ currents or by prolonged APs and refractoriness, leading to shorter repolarisation phase for Na^+^ recovery from inactivation.

Taken together, these results demonstrate that the robust shape of the optogenetically paced FP allows the detection of drug effects on three main cardiac ion channels, which could be quantified automatically due to the characteristic changes within the FP.

### 2.5. Evaluation of Drug Screening with the CardioExcyte96 System

**Figure 5:**
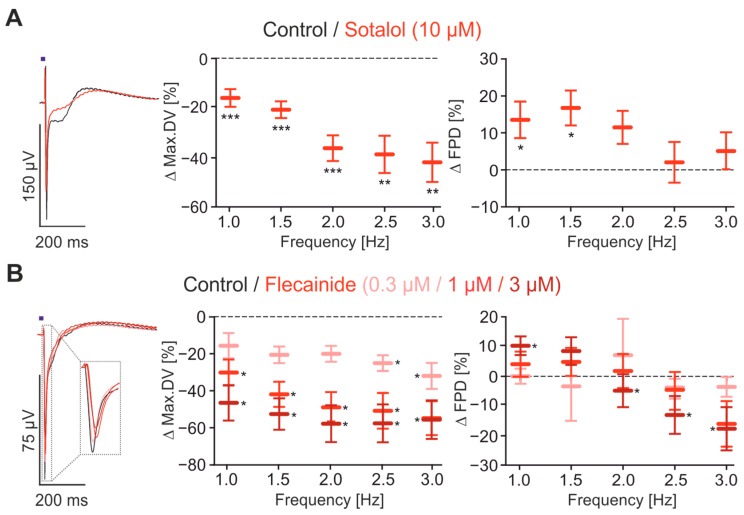
Analysis of drug effects on FP. (**A**,**B**) Overlay of representative FP optical paced at 1 Hz (**left**) before (black) and after drug application (red), and statistical analysis of the relative changes of max.DV (**middle**) and FPD (**right**) at the frequencies 1–3 Hz. (**A**) Effects of 10 μM Sotalol (*n* = 13–17); (**B**) effects of 0.3, 1 and 3 μM Flecainide (*n* = 11–12). * *p* < 0.05, ** *p* < 0.01, *** *p* < 0.001.

To assess if our approach is suitable for cardiotoxic drug screening, we next tested the effect of known arrhythmogenic substances. First, we investigated the β-blocker Sotalol, which is known to block also hERG K^+^ channels and thereby induce a pharmacological Long QT syndrome with the high risk of arrhythmia [33,46]. The application of 10 μM of Sotalol significantly prolonged the FPD but only at the low frequencies of 1 and 1.5 Hz, and not at higher frequencies (Figure 5A). In addition, Sotalol significantly reduced the max.DV with strongest effects at frequencies >2 Hz (Figure 5A middle).

To test if our system detects side effects on Na^+^ channels that could lead to drug-induced Brugada syndrome [5,47], we applied the class Ic anti-arrhythmic drug Flecainide, a blocker for the fast Na^+^ current, which significantly reduced the max.DV parameter in a dose-dependent manner (Figure 5B). Interestingly, high concentrations of Flecainide (3 μM) also affected the FPD in a frequency-dependent manner. At low pacing rates (1 Hz), the FPD was significantly prolonged, whereas at high rates (>2 Hz) the FPD was clearly reduced (Figure 5B right).

**Figure 6:**
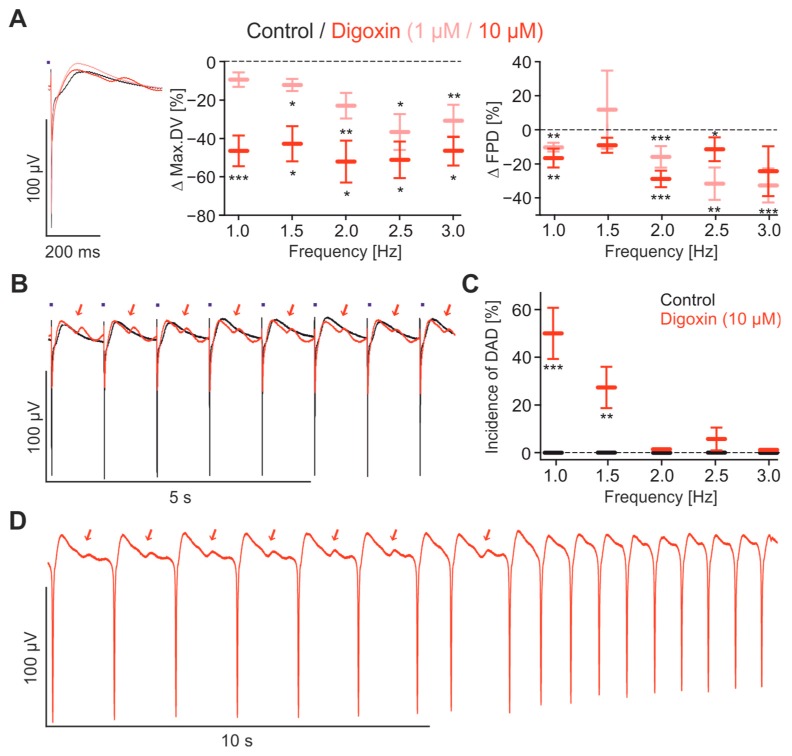
Effects of Digoxin. (**A**) Overlay of representative FP optically paced at 1 Hz (**left**) before (black) and after Digoxin application (red), and statistical analysis of the effects of 1 μM and 10 μM Digoxin on relative changes of max.DV (**middle**) and FPD (**right**) at the frequencies 1–3 Hz (*n* = 10–11); (**B**) representative FP traces paced at 1 Hz in control conditions (black) and after application of 10 μM Digoxin (red) with arrhythmic afterdepolarisations (red arrows); (**C**) statistical analysis of the incidence of afterdepolarisations at pacing rates of 1–3 Hz (*n* = 15); (**D**) FP trace from a spontaneously beating monolayer the presence of 10 μM Digoxin. Note the afterdepolarisations (red arrows) after each FP in the first phase (**left**) and the double beating rate in the second phase (**right**) indicating a FP triggering by each afterdepolarisation * *p* < 0.05, ** *p* < 0.01, *** *p* < 0.001.

It is known that cardiac glycosides can cause arrhythmias [48,49,50], through the indirect blocking of the Na^+^/Ca^2+^ exchanger and Ca^2+^ accumulation. To test for such effects, we applied Digoxin at two concentrations (1 and 10 μM), which consistently reduced the max.DV in a dose-dependent manner and shortened the FPD at almost all beating rates (Figure 6A). In particular, the latter effect is in accordance with recent studies [51], and reports demonstrating that Digoxin can shorten the QT duration in the ECG [49,50] and that this could be due to Ca^2+^-dependent inactivation of L-Type Ca^2+^-channels [52]. Importantly, in the presence of 10 μM Digoxin, we observed afterdepolarisations occurring at the end of the repolarisation phase of the FP (Figure 6B). Interestingly the afterdepolarisations occurred only at low pacing rates (<1.5 Hz) and were not seen at higher rates (Figure 6C). We speculate that these signals result from spontaneous Ca^2+^ release events which are thought to be responsible for arrhythmias induced by intoxication of cardiac glycosides [48]. Importantly, the rate dependency of the proarrhythmic effect of Digoxin has also been observed in AP and ECG recordings [53]. Interestingly, in the presence of 10 μM Digoxin one FP recording from spontaneously beating cardiomyocytes showed a transition from a state characterized by regular afterdepolarisations to a state without afterdepolarisations, but with twice the beating rate, suggesting that in the latter state each afterdepolarisation triggers an AP (Figure 6D).

### 2.6. Xpress.4U^TM^ LightPace for Virus Free ChR2 Expression

**Figure 7:**
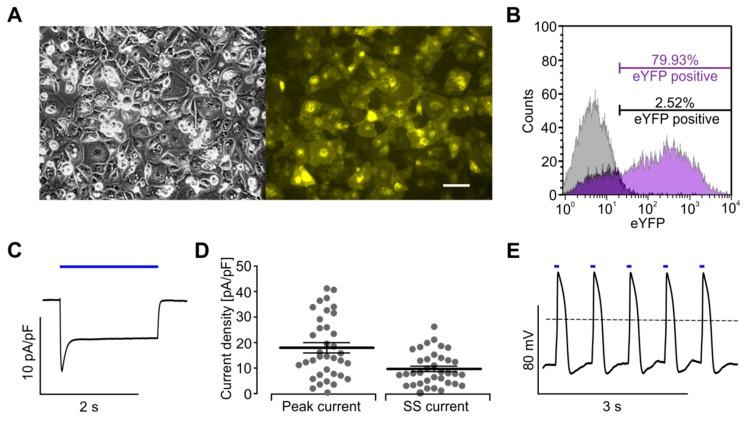
Characterisation of Xpress4U^TM^ LightPace transfected hiPSC-derived Cor.4U^®^ cardiomyocytes. (**A**) Phase contrast (**left**) and fluorescence enhanced yellow fluorescent protein (EYFP) image (**right**) 17 h after Xpress.4U^TM^ LightPace transfection (scale bar: 100 μm); (**B**) FACS analysis profile for ChR2-EYFP from Xpress4U^TM^ LightPace transfected cells (purple) compared to non-infected control cells (grey); (**C**,**D**) light-induced (3.0 mW/mm^2^) inward current at a holding potential of −40 mV in a Xpress4U^TM^ LightPace transfected cell (**C**) and statistics of ChR2 peak currents and steady state (SS) currents (**D**, *n* = 36 cells); (**E**) representative trace of action potentials (APs) evoked by light pulses (10 ms, 3.0 mW/mm^2^, blue bars).

To avoid the virus-mediated gene transfer of ChR2 in hiPSC-derived cardiomyocytes, which are difficult to transfect by standard methods, we took advantage of the commercially available Xpress.4U^TM^ LightPace kit (Ncardia). This method is based on special lipid particles containing mRNA for the expression of ChR2 and a C-terminal enhanced yellow fluorescent protein (EYFP)-tag. Already, 17 h after transfection, the expression of ChR2-EYFP was clearly detectable by fluorescence microscopy in over 70% of cells in a confluent monolayer (Figure 7A). Adverse side effects from the transfection procedure or ChR2-EYFP expression could not be observed (Figure 7A). FACS analysis of dissociated cells quantified 79.9% EYFP positive cells, the same FACS setting detected 2.5% cells in untreated control cells (Figure 7B). In order to quantify the ChR2 function, Xpress4U^TM^ LightPace-treated cells were analysed using the patch clamp technology. We detected large light-induced inward currents (Figure 7C) with an average peak current of 17.9 ± 2.0 pA/pF and a steady state current of 9.6 ± 1.1 pA/pF (Figure 7D, *n* = 36). The level of ChR2 expression was sufficient for optical pacing (Figure 7E) at 1.5 Hz using 10 ms long light pulses of 3.0 mW/mm^2^ in 86.4% of cells (*n* = 38). Thus, the Xpress.4U^TM^ LightPace technology is well suited for functional ChR2 expression and is an easy to handle, non-viral and commercially available tool for the optogenetic pacing of hiPSC-derived cardiomyocytes.

### 2.7. Future Trend and Automatisation with CardioExcyte96 SOL

**Figure 8:**
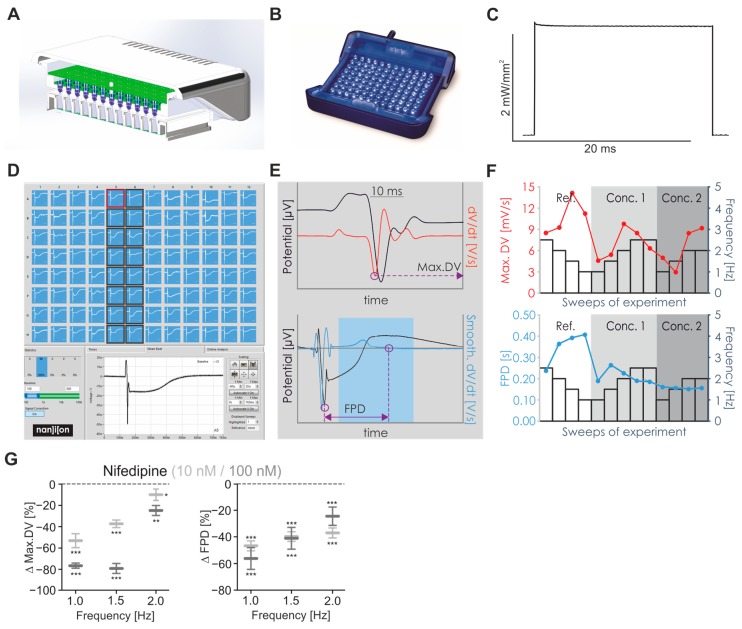
Automated FP recording and analysis of Xpress4U^TM^ LightPace transfected cells with CardioExcyte96 SOL platform. (**A**) Sketch of the CardioExcyte96 SOL with 96 LEDs integrated in the lid of a temperature controlled incubation system; (**B**) picture of the CardioExcyte96 SOL lid; (**C**) light intensity profile of a 20 ms light pulse from one LED at the level of recording electrodes; (**D**–**F**) workflow of the CardioExcyteControl analysis software; (**D**) screenshot showing optogenetically paced and averaged FP from all 96 wells; (**E**) description of automated analysis routines. For the max.DV parameter (**E**, **top**), the first derivative (red) is calculated from the first phase (60 ms window) of the FP (black) and its minimum (violet circle) is defined as max.DV. The FPD (**E**, **lower**) is defined from the minimal time point (first violet circle) in the FP signal (black) to the time point within a customizable analysis window (shaded blue) at which the smoothed first derivative (blue) is zero (second violet circle); (**F**) the values for max.DV (**top**) and FPD (**bottom**) are plotted for each recording episode with different pacing frequencies (1–2 Hz, indicated by bar graphs) and in different conditions (“Ref”: baseline recordings, “Conc. 1”: 10 nM Nifedipine, “Conc. 2” = 100 nM Nifedipine); (**G**) relative changes of max.DV and FPD by application of 10 nM and 100 nM Nifedipine (*n* = 16). * *p* < 0.05, ** *p* < 0.01, *** *p* < 0.001.

To demonstrate our approach with commercially available products and fully automated procedures, we developed the CardioExcyte96 SOL platform (Figure 8A,B) and integrated it into the existing CardioExcyteControl recording software. This new commercially available LED lid is based on the above-described prototype and enables precisely controlled light pulses at durations from 1 to 50 ms with a rise time <40 μs, and a light intensity of 2.0 mW/mm^2^ on each recording electrode (Figure 8C). Thereby, the Xpress.4U^TM^ LightPace transfected hiPSC-derived cardiomyocytes could be paced efficiently at beating rates from 1 to 2 Hz. As proof of principle for automated cardiotoxicity screening, we tested the effect of Nifedipine with recording and analysis procedures performed by the CardioExcyteControl software. The workflow includes optical pacing and recording of evoked FP from 96 wells simultaneously (Figure 8D) and the subsequent automated analysis of max.DV and FPD (Figure 8E,F). The results showed reduction of max.DV and FPD by Nifedipine in a dose-dependent manner at all pacing frequencies (Figure 8G), similarly to the above-described and manually analysed results on AAV-transfected cardiomyocytes (Figure 4C).

## 3. Discussion

In this report, we demonstrate that combining optogenetic stimulation with multi-well FP recordings from hiPSC-derived cardiomyocytes enables fully automated cardiotoxicity screening. Importantly, our approach is based on simple, reproducible and easy-to-train protocols and all components are commercially available. We show two different strategies to express ChR2 in the hiPSC-derived cardiomyocytes, which could be both parallelized by standard liquid handling robots. Gene transfer via AAV is very effective even when using tenfold lower virus titer in comparison to our earlier report [17] without decreased expression rate or reduced pacing efficiency. Importantly, expression was very stable over time and allowed optogenetic pacing even four weeks after transduction, which is in line with reliable AAV-based ChR2 gene transfer of mouse hearts in vivo more than one year after virus injection [54,55]. Therefore this approach will also allow to detect chronic effects of drugs, which promote arrhythmia only after long-term application, such as tyrosine kinase inhibitors used in cancer therapy and some hERG inhibitors also affecting the late Na^+^ current [56,57]. For such long-term experiments the new CardioExcyte96 SOL device is equipped with an incubator system which enables optogenetic pacing and FP recording at freely chosen intervals. For users who would like to circumvent virus handling, the new Xpress.4U^TM^ LightPace kit from Ncardia is an alternative. After a simple sonication step to generate fusogenic liposomes containing mRNA, this kit allows the expression of ChR2 in fusion with EYFP within hours after transfection of cells. In the future, the Xpress.4U™ technology could also be used for other genetic modifications. Specifically for the cardiac arrhythmia field, CRISPR/Cas9-based induction or correction of single mutations [58] in combination with patient specific hiPSC-derived cardiomyocytes would allow elegant studies investigating the effects of genetic Long QT, Short QT or Brugada syndromes.

In our report, cardiomyocytes expressing ChR2 using Xpress.4U^TM^ LightPace transfection required longer light pulses (10–20 ms) for reliable pacing than AAV-based ChR2 expression (1–2 ms) when using similar light intensities. Compared to our recent analysis of ChR2 mediated inward currents in AAV transduced hiPSC-derived cardiomyocytes [17], we found in average comparable light-induced inward currents after expressing ChR2 using the Xpress.4U^TM^ ChR2 mRNA transfection, but also much higher variability between cells (Figure 7D). Because cardiomyocytes are electrically coupled through gap junctions when plated on MEA electrodes as a monolayer, the high variability of ChR2 current might explain the overall lower excitability of Xpress.4U^TM^ LightPace transfected cells. Importantly, both the AAV and the Xpress.4U^TM^ LightPace strategies allowed pacing with non-toxic low intensity light, which can be generated by standard LEDs. Importantly, the design of our initial custom-built LED lid and our analysis algorithms were integrated in a new commercially available optogenetic screening system, including a temperature-controlled LED lid with incubator function as well as an integrated FP recording and LED stimulation software (CardioExcyte96 SOL, Nanion Technologies).

The exact control of and measurement at a specific beating rate is very important in order to detect rate-dependent alterations of APD by drugs or mutations. Because of the limited range of spontaneous beating rates of hiPSC-derived cardiomyocytes under physiological conditions as well as because of a possible modulation of the beating rate by applied drugs, rate-dependent effects could be missed when measuring only at spontaneous beating rates and correcting the FPD with non-validated QT_c_ correction formulas [22,24,59]. Using optogenetic stimulation, the hERG channel blocker E4031 and the drug Sotalol showed the most FPD prolonging effect at lower heart rates. This is in accordance with the reverse use dependence of most hERG blockers [33], and with clinical studies on drug-induced Long QT syndromes reporting the induction of malignant Torsades de Pointes arrhythmia, especially at slow beating rates or by the first beat after a long post-extrasystolic pause [2]. 

We have previously shown that optogenetic pacing in vitro by global illumination results in a simultaneous activation of all cardiomyocytes and electrical activity without a conduction delay [17]. This advantage becomes evident when recording FP with large recording electrodes such as those in the CardioExcyte96 system with a 2 mm diameter. Due to the fact that, in our approach, one LED is illuminating the whole recording electrode, the simultaneous activation of all cardiomyocytes is physically averaged resulting reliably in one FP with the typical shape. The signal-to-noise ratio and thus quality of the FP recordings is dependent on the amount of cells from which the electrical activity can be recorded. In principle, our approach could be expanded to even larger electrodes as long as sufficient global illumination is feasible. The simultaneous activation is especially important for the initial component of the FP, because only one continuous and non-fragmented fast downstroke signal allows the analysis of the max.DV parameter, which reflects the fast Na^+^ and Ca^2+^ current-based depolarisation during phase 0 of the cardiac AP. Thus optogenetic pacing of cardiomyocytes on large electrodes also enables drug screening for side effects slowing the AP depolarisation, either by directly blocking Na^+^ or Ca^2+^ currents or indirectly by elevating the resting membrane potential leading to voltage-dependent inhibition of Na^+^ currents. Because such effects are slowing electrical conduction and are therefore highly pro-arrhythmogenic, detecting drug action on the max.DV parameter adds great value to a cardiotoxicity screening system. Likewise, we detected not only the well-known effect of Sotalol on FP prolongation, but also its negative side effect on the AP depolarisation phase, which has previously been described [60,61]. 

The need for frequency-dependent drug screening becomes even more evident when analysing the effects of the Na^+^ channel blocker Flecainide, which not only showed the expected reduction of the max.DV parameter but also prolonged the FP only at low beating rates. This was shown previously [62] and can be explained by blocking of hERG channels [63]. However, at a high pacing frequency, we detected a significant and concentration-dependent shortening of the FP. Together with the pronounced inhibitory effect on the max.DV parameter particularly at fast beating, this could be due to an inhibitory side effect on Ca^2+^ channels or due to a blocking effect on the late Na^+^ current. In particular, the latter effect was discussed before to be more effective at higher rates [64] when hERG currents have less influence on repolarisation. In addition to the specific FPD and max.DV parameters, which can be considered as an indirect measure of pro-arrhythmogenic side effects of drugs, we were able to detect afterdepolarisation-like signals in FP recordings after treating cardiomyocytes with Digoxin, but only at low pacing rates. Because such events are likely to be a key trigger to induce a cardiac arrhythmia, this analysis adds another important and direct surrogate parameter for pro-arrhythmia drug screening, which is one of the main goals of the CiPA initiative [9]. 

## 4. Materials and Methods 

### 4.1. Cell Culture and AAV-Mediated Transduction 

For each batch a total of 1 × 10^6^ hiPSC-derived cardiomyocytes (Cor.4U^®^ cells, Ncardia) were ordered and delivered non-frozen in a T25 flask. On the day of arrival, the cells were transduced with 6550 genome copies/cell of AAV to express ChR2 (PennVector Core, University of Pennsylvania, PA, USA). We used the AAV 2.1 (AAV serotype 2 with capsid of serotype 1) with ChR2 in fusion with the fluorescence protein mCherry under control of the CAG-promotor for high expression in cardiomyocytes. After two days of incubation at 37 °C and 5% CO_2_ in Cor.4U^®^ medium, the cells were dissociated as reported previously [17]. In brief, the cells were incubated in the flask with TrypLE Express (Thermo Fisher Scientific, Hennef, Germany) at 37 °C and 5% CO_2_ for 4 min. After adding of 10 mL of ice-cold Cor.4U^®^ medium, cells were detached and incubated at 4 °C for 20 min and then centrifuged at 700 rpm for 4 min before being finally resuspended and used for downstream analysis.

### 4.2. Determination of ChR2-Expression after AAV-Transduction

For quantification of ChR2-expression three weeks after AAV-based gene transfer by FACS analysis, 100,000 transduced Cor.4U^®^ cells were plated per well in fibronectin-coated (10 μg/mL; Sigma Aldrich, Darmstadt, Germany) 24-well plates and cultured in Cor.4U^®^ medium (changed twice a week). After three weeks, cells were dissociated with 1 mL TrypLE Express dissolved in 1 mL phosphate-buffered saline (PBS) and analysed with a CyFlow Space FACS (Partec GmbH, Görlitz, Germany) equipped with a 561 nm laser for excitation and a 590 ± 50 nm emission filter. Cells positive for mCherry were quantified by determining the gating setting using mCherry negative cells. 

For immunostaining, cells were dissociated as above and plated on fibronectin coated cover glass and cultured for three weeks. After fixation with 4% paraformaldehyde (PFA), the cells were stained with primary antibodies against cardiac Troponin T (Abcam, Cambridge, UK) in 0.2% Triton X (Sigma Aldrich) and 5% Donkey Serum for 1.5 h and Cy2-conjugated antibody against rabbit ImmunglobulinG (Thermo Fisher Scientific) for 1 h in Hoechst (1 μg/mL in PBS). Microscope pictures were taken with an inverted fluorescence microscope (Axiovert 200 M, Zeiss, Oberkochen, Germany) using the Apotome section module, through a 40× Plan-Apochromat objective (Zeiss), mCherry (F46-008), Cy2 (HC 470-22ex, HC 495bs, HC 510/10em) filter sets (AHF Analysentechnik, Tübingen, Germany) and the AxioCam MRm camera with the AxioVision software (Version 4.8.2, Zeiss). 

### 4.3. CardioExcyte96 LED Setup and FP Recordings

For FP recording, cardiomyocytes were plated directly on recording electrodes of CardioExcyte sensor plates (NSP-96, Nanion Technologies). For this purpose, cells were dissociated as described above, centrifuged and resuspended to obtain 20,000 cells per 2 μL drop. Recording electrodes were coated with 2 μL fibronectin (10 μg/mL; Sigma Aldrich) for 30–45 min at 37 °C and 5% CO_2_. Subsequently the fibronectin solution was removed and a 2 μL drop of the cell suspension was placed exactly on the recording electrode. After 45 min incubation at 37 °C and 5% CO_2_, the wells were gently filled with 300 μL of Cor.4U^®^ medium. After plating locally on the recording electrode, the cardiomyocytes formed a confluent monolayer which started to beat spontaneously. Plates were incubated for three to four weeks at 37 °C and 5% CO_2_. Twice a week, approximately two-thirds of the medium was exchanged to reduce sheer stress and to prevent cell loss.

FP were recorded from the CardioExcyte sensor plates with the CardioExcyte96 system (Nanion Technologies). For application of light pulses, a LED lid was custom built with 96 LEDs (470 nm, NSPB500AS, Nichia). LEDs were powered by constant current triggered LED drivers (LUMITRONIX, Hechingen, Germany) at 100 mA. The light intensity was calibrated with a fast powermeter (PM100A with S170C sensor, Thorlabs, Newton, NJ, USA) placed at the level of the recording electrodes and a pinhole of the size of a recording electrode. 

FP were recorded in 20 s long sweeps at 10 kHz sampling rate at 35 °C and 5% CO_2_ in IMDM medium without FCS containing 1% non-essential amino acids (Gibco, Thermo Fisher Scientific), 1% penicillin/streptomycin (Gibco, Thermo Fisher Scientific) and 0.1% β-mercaptoethanol (Sigma Aldrich). After recording of baseline control signals, compounds were added in the indicated concentrations from stock solutions (Digoxin 1 mM in MeOH, E4031 1 mM in Iscove’s Modified Dulbecco’s Media (IMDM), Isoprenaline 1 mM in IMDM, Lidocaine 100 mM in EtOH, Nifedipine 3 mM in EtOH, Sotalol 10 mM in IMDM).

### 4.4. Analysis of FP

All individual FP in the 20 s long recording sweeps were averaged using the time point and the frequency of LED stimulation as the averaging trigger. The max.DV parameter and the FPD were determined manually (Figure 2, Figure 3, Figure 4, Figure 5 and Figure 6) using the Origin Pro 8G software (Version 8.0988, OriginLab, Northampton, MA, USA) or automatically with the CardioExcyteControl recording software (Figure 8). Some wells showed inverted FP signals with a fast positive initial component and negative repolarisation wave, and these data were inverted before subsequent analysis. The max.DV parameter was defined as the most negative value of the first derivative of the FP. For determining the FPD, the delay from the first negative spike of the FP to the time point at which the smoothed (Savitzky-Golay filter, polynumeric order = 2, window = 500 data points) first derivative of the FP was zero (see also Figure 8E). Afterdepolarisations were defined as clearly visible negative deflections occurring in the repolarisation phase of the FP and quantified in non-averaged recording sweeps by calculation of the percentage of FP with afterdepolarisations. 

### 4.5. Determination of ChR2-Expression in Xpress.4U^TM^ LightPace Transfected Cor.4U^®^ Cells 

Cor.4U^®^ cells were seeded on Geltrex-coated T-25 flasks (1 × 10^6^ cells/flask) and cultured for three days in Cor.4U^®^ medium (changed daily). On day 3, cells were transfected using the Xpress.4U^TM^ LightPace kit for ChR2 expression (Ncardia) according to the manufacturer’s instructions. Then, 17 h after transfection, microscopic images were acquired using an epifluorescence microscope (Axiovert 200M, Zeiss), equipped with a 10× Achroplan (Ph1, NA 0.25, Zeiss), a YFP filter set (F41-028, AHF Analysetechnik), a HBO 100 mercury arc lamp and a 1.4 MP cooled digital camera (SPOT Pursuit, Diagnostics Instruments Inc., Burroughs, MI, USA). After image acquisition, cells were detached from T-25 flasks by Trypsin/Ethylenediaminetetraacetic acid (EDTA) (Sigma Aldrich) treatment for 5 min at 37 °C and centrifuged at 200× *g* for 5 min. Cell pellets were resuspended in PBS containing 2% fetal calf serum (FCS) and filtered with a 40 μm cell strainer to obtain a uniform single-cell suspension for flow cytometric analyses (BD FACS Calibur, BD Biosciences, Singapore) equipped with 488 nm argon laser for excitation and a 530/30 nm FL1 emission filter.

### 4.6. Patch Clamp Analysis of Xpress.4U^TM^ LightPace Transfected Cor.4U^®^ Cells 

For patch clamp analysis, Cor.4U^®^ cells were seeded in a T-25 flask and transfected with the Xpress.4U^TM^ LightPace kit for ChR2 expression (Ncardia) in 2 mL volume for 30 min at 37 °C accordingly to the manufacturer’s instructions. Cor.4U^®^ medium was added and cells were incubated for 8 h. Subsequently cells were dissociated from the T-25 flask as described above and replated at low densities on fibronectin-coated (10 μg/mL) coverslips. Patch clamp experiments were performed two to three days after mRNA transfection in the whole cell configuration using an EPC 10 amplifier (HEKA Elektronik, Ludwigshafen, Germany). The internal solution contained (in mmol/L) 50 KCl, 80 K-Aspartate, 1 MgCl_2_, 3 MgATP, 10 Ethylene-bis(oxyethylenenitrilo)tetraacetic acid (EGTA), 10 4-(2-Hydroxyethyl)piperazine-1-ethanesulfonic acid, *N*-(2-Hydroxyethyl) piperazine-*N*′-(2-ethanesulfonic acid) (HEPES), pH 7.2 (KOH) and the external solution 140 NaCl, 5.4 KCl, 1.8 CaCl_2_, 1 MgCl_2_, 10 HEPES, 10 Glucose, pH 7.4 (NaOH). Cells were stimulated with blue light (470 nm) from a LED module (LEDHUB, Omicron Laserage, Rodgau, Germany) coupled to the epifluorescence port of an Axiovert 200 microscope (Zeiss) and controlled by the EPC 10 amplifier. Light-induced currents were characterised in the voltage clamp mode at a holding potential of −40 mV with supramaximal light stimulation of 3.0 mW/mm^2^ for 2 s. Peak currents were analysed by determining the maximal inward current and the steady-state current was averaged between 1320 to 1920 ms after start of the light pulse using Fitmaster (HEKA Elektronik). AP were recorded in the current clamp mode and were elicited by 10 ms-long blue light pulses (470 nm) at increasing light intensities (0–3 mW/mm^2^). Light stimulation was repeated five times at a frequency of 1.5 Hz, and the minimal light intensity for 100% AP generation was determined. 

### 4.7. FP Recordings of Xpress.4U^TM^ LightPace Transfected Cor.4U^®^ Cells with the CardioExcyte96 SOL System

For FP recording, 30,000 wild-type Cor.4U^®^ cells were plated on CardioExcyte sensor plates as described above and transfected at day 10 with the Xpress.4U^TM^ LightPace kit for ChR2 expression (50 μL final volume per well of the CardioExcyte sensor plate) for 30 min at 37 °C. Recording of optogenetically paced FP was performed two days after mRNA transfection on the commercially available CardioExcyte96 SOL system (Nanion Technologies). The light intensity of the CardioExcyte96 SOL was determined, as described above. Automated analysis of max.DV and FPD was performed with CardioExcyteControl software as described above (see also Figure 8D and its legend). The analysis window to determine FPD is customizable as a fraction of the inter-beat interval (1/pacing rate) to account for different FP shapes and the frequency-dependent FPD.

### 4.8. Statistical Analysis

All data is shown as mean ± SEM. Values of max.DV and FPD are either presented as absolute values (Figure 2 and Figure 3C,D) or as the percent change normalized to control conditions without drug application (Figure 3E, Figure 4, Figure 5, Figure 6 and Figure 8). Statistical analysis on drug effects was performed with two-sided paired student’s *t*-test by comparing FP parameters in the presence of drugs to baseline conditions for each pacing frequency individually. The FP alteration over time (Figure 2) and the frequency dependence of FP parameters (Figure 3C,D) were analysed with one-way ANOVA repeated measurements and Dunnett’s post-test. A *p* value of <0.05 was considered statistically significant and the n-values are indicating the number of independent wells of the CardioExcyte sensor plates. Statistical analysis was performed using GraphPad Prism (Version 5.01, GraphPad Software, La Jolla, CA, USA).

## 5. Conclusions

We herein present a fully automated approach, which enables frequency dependent cardiotoxicity screening in a scalable 96-well format. Importantly, the CardioExcyte96 SOL optogenetic drug screening system is available as an all-in-one tool including the software needed for automated recording and analysis (Figure 8). Our approach allows the determination of rate-dependent side effects of drugs on K^+^, Na^+^ and Ca^2+^ channels as surrogate parameters for cardiac arrhythmia propensity and therefore represents an important step towards scalable cardiotoxicity screening and will increase safety and effectiveness of drug screening in the pharmaceutical industry.

## Abbreviations

AAV	adeno-associated virus
AP	action potential
APD	action potential duration
CAG	chicken β actin
ChR2	Channelrhodopsin-2
CiPA	comprehensive in vitro proarrhythmia assay
ECG	electrocardiogram
EDTA	Ethylenediaminetetraacetic acid
EGTA	Ethylene-bis(oxyethylenenitrilo)tetraacetic acid
EYFP	enhanced yellow fluorescent protein
FACS	fluorescence-activated cell sorting
FCS	fetal calf serum
FP	field potential
FPD	field potential duration
hERG	human Ether-a-go-go Related Gene
HEPES	4-(2-Hydroxyethyl)piperazine-1-ethanesulfonic acid, *N*-(2-Hydroxyethyl)piperazine-Nesulfonic idhors de acid)
hiPSC	human induced pluripotent stem cells
IMDM	Iscove’s Modified Dulbecco’s Media
I_Kr_	rapid potassium current
LED	Light-emitted diode
max.DV	maximum negative downstroke velocity
MEA	microelectrode array
PBS	Phosphate-buffered saline
PFA	paraformaldehyde
