# Correction: Rehnelt et al. Frequency-Dependent Multi-Well Cardiotoxicity Screening Enabled by Optogenetic Stimulation. *Int. J. Mol. Sci.* 2017, *18*, 2634

**DOI:** 10.3390/ijms22115562

**Published:** 2021-05-25

**Authors:** Susanne Rehnelt, Daniela Malan, Krisztina Juhasz, Benjamin Wolters, Leo Doerr, Matthias Beckler, Ralf Kettenhofen, Heribert Bohlen, Tobias Bruegmann, Philipp Sasse

**Affiliations:** 1 Institute of Physiology I, Medical Faculty, University of Bonn, 53127 Bonn, Germany; s4surehn@uni-bonn.de (S.R.); dmalan@uni-bonn.de (D.M.); 2 Nanion Technologies GmbH, 80636 Munich, Germany; krisztina.juhasz@nanion.de (K.J.); leo.doerr@nanion.de (L.D.); matthias.beckler@nanion.de (M.B.); 3 Part of the Ncardia Group, Axiogenesis AG, 50829 Cologne, Germany; benjamin.wolters@ncardia.com (B.W.); ralf.kettenhofen@ncardia.com (R.K.); heribert.bohlen@ncardia.com (H.B.); 4 Research Training Group 1873, University of Bonn, 53127 Bonn, Germany

The authors wish to make the following corrections to this paper [1]:

The commercial human cardiomyocytes used in the study (Cor.4U©, NCardia/Axiogenesis AG, Cologne, Germany) were believed to be derived from human induced pluripotent stem cells during the studies. After the studies had been completed and published, short tandem repeat testing by NCardia determined that Cor.4U© cardiomyocytes have the genetic background of the human embryonic stem cell line RUES2. The different provenience of these well-characterized cardiomyocytes changes neither our findings nor the interpretation of our data.

The authors would like to apologize for any inconvenience caused to the readers by these changes.
